# Supplementary material for: HIVprotI: an integrated web based platform for prediction and design of HIV proteins inhibitors
Source: J Cheminform. 2018 Mar 9;10:12. doi: 10.1186/s13321-018-0266-y (PMC5845081; doi:10.1186/s13321-018-0266-y)
Supplement: Supplementary file 2 — Additional file 2. Source code of HIVProtI web server. [file 13321_2018_266_MOESM2_ESM.zip › HIVprotI_Source-code/hivprotI/weka-3-6-6/doc/weka/experiment/DatabaseResultListener.html]

DatabaseResultListener


---


|  |  |  |
| --- | --- | --- |
| **Overview** \| **Package** \| **Class** \| **Tree** \| **Deprecated** \| **Index** \| **Help** \| **Weka's home** | |  |
| **PREV CLASS**   **NEXT CLASS** | **FRAMES**    **NO FRAMES**     **All Classes** |  |
| SUMMARY: NESTED \| FIELD \| CONSTR \| METHOD | DETAIL: FIELD \| CONSTR \| METHOD |  |


---


## weka.experiment Class DatabaseResultListener

```
java.lang.Object
  weka.experiment.DatabaseUtils
      weka.experiment.DatabaseResultListener
```

**All Implemented Interfaces:**: java.io.Serializable, RevisionHandler, ResultListener

**Direct Known Subclasses:**: DatabaseResultProducer

---

``` public class DatabaseResultListener extends DatabaseUtils implements ResultListener ```

Takes results from a result producer and sends them to a database.

**Version:**
:   $Revision: 5125 $

**Author:**
:   Len Trigg (trigg@cs.waikato.ac.nz)

**See Also:**: Serialized Form

---

**Field Summary**

**Fields inherited from class weka.experiment.DatabaseUtils**

`BOOL, BYTE, DATE, DOUBLE, EXP_INDEX_TABLE, EXP_RESULT_COL, EXP_RESULT_PREFIX, EXP_SETUP_COL, EXP_TYPE_COL, FLOAT, INTEGER, LONG, PROPERTY_FILE, SHORT, STRING, TEXT, TIME`


**Constructor Summary**

`DatabaseResultListener()`
  
          Sets up the database drivers


| **Method Summary** | |
| --- | --- |
| `void` | `acceptResult(ResultProducer rp, java.lang.Object[] key, java.lang.Object[] result)`             Submit the result to the appropriate table of the database |
| `java.lang.String` | `cacheKeyNameTipText()`             Returns the tip text for this property |
| `java.lang.String[]` | `determineColumnConstraints(ResultProducer rp)`             Determines if there are any constraints (imposed by the destination) on any additional measures produced by resultProducers. |
| `java.lang.String` | `getCacheKeyName()`             Get the value of CacheKeyName. |
| `java.lang.String` | `getRevision()`             Returns the revision string. |
| `java.lang.String` | `globalInfo()`             Returns a string describing this result listener |
| `boolean` | `isResultRequired(ResultProducer rp, java.lang.Object[] key)`             Always says a result is required. |
| `void` | `postProcess(ResultProducer rp)`             Perform any postprocessing. |
| `void` | `preProcess(ResultProducer rp)`             Prepare for the results to be received. |
| `void` | `setCacheKeyName(java.lang.String newCacheKeyName)`             Set the value of CacheKeyName. |

**Methods inherited from class weka.experiment.DatabaseUtils**

`arrayToString, close, close, connectToDatabase, createExperimentIndex, createExperimentIndexEntry, createResultsTable, databaseURLTipText, debugTipText, disconnectFromDatabase, execute, experimentIndexExists, getDatabaseURL, getDebug, getKeywords, getKeywordsMaskChar, getPassword, getResultFromTable, getResultSet, getResultsTableName, getSupportedCursorScrollType, getUsername, isConnected, isCursorScrollable, isCursorScrollSensitive, isKeyword, maskKeyword, passwordTipText, processKeyString, putResultInTable, select, setDatabaseURL, setDebug, setKeywords, setKeywordsMaskChar, setPassword, setUsername, tableExists, translateDBColumnType, typeName, update, usernameTipText`

**Methods inherited from class java.lang.Object**

`equals, getClass, hashCode, notify, notifyAll, toString, wait, wait, wait`

**Constructor Detail**

### DatabaseResultListener

```
public DatabaseResultListener()
                       throws java.lang.Exception
```

:   Sets up the database drivers

    **Throws:**: `java.lang.Exception` - if an error occurs


**Method Detail**

### globalInfo

```
public java.lang.String globalInfo()
```

:   Returns a string describing this result listener

    :   **Returns:**: a description of the result listener suitable for displaying in the explorer/experimenter gui

---


### preProcess

```
public void preProcess(ResultProducer rp)
                throws java.lang.Exception
```

:   Prepare for the results to be received.

    :   **Specified by:**: `preProcess` in interface `ResultListener`
    :   **Parameters:**: `rp` - the ResultProducer that will generate the results **Throws:**: `java.lang.Exception` - if an error occurs during preprocessing.

---


### postProcess

```
public void postProcess(ResultProducer rp)
                 throws java.lang.Exception
```

:   Perform any postprocessing. When this method is called, it indicates
    that no more results will be sent that need to be grouped together
    in any way.

    :   **Specified by:**: `postProcess` in interface `ResultListener`
    :   **Parameters:**: `rp` - the ResultProducer that generated the results **Throws:**: `java.lang.Exception` - if an error occurs

---


### determineColumnConstraints

```
public java.lang.String[] determineColumnConstraints(ResultProducer rp)
                                              throws java.lang.Exception
```

:   Determines if there are any constraints (imposed by the
    destination) on any additional measures produced by
    resultProducers. Null should be returned if there are NO
    constraints, otherwise a list of column names should be
    returned as an array of Strings. In the case of
    DatabaseResultListener, the structure of an existing database
    will impose constraints.

    :   **Specified by:**: `determineColumnConstraints` in interface `ResultListener`
    :   **Parameters:**: `rp` - the ResultProducer to which the constraints will apply **Returns:**: an array of column names to which resutltProducer's results will be restricted. **Throws:**: `java.lang.Exception` - if an error occurs.

---


### acceptResult

```
public void acceptResult(ResultProducer rp,
                         java.lang.Object[] key,
                         java.lang.Object[] result)
                  throws java.lang.Exception
```

:   Submit the result to the appropriate table of the database

    :   **Specified by:**: `acceptResult` in interface `ResultListener`
    :   **Parameters:**: `rp` - the ResultProducer that generated the result: `key` - The key for the results.: `result` - The actual results. **Throws:**: `java.lang.Exception` - if the result couldn't be sent to the database

---


### isResultRequired

```
public boolean isResultRequired(ResultProducer rp,
                                java.lang.Object[] key)
                         throws java.lang.Exception
```

:   Always says a result is required. If this is the first call,
    prints out the header for the Database output.

    :   **Specified by:**: `isResultRequired` in interface `ResultListener`
    :   **Parameters:**: `rp` - the ResultProducer wanting to generate the result: `key` - The key for which a result may be needed. **Returns:**: true if the result should be calculated. **Throws:**: `java.lang.Exception` - if the database couldn't be queried

---


### cacheKeyNameTipText

```
public java.lang.String cacheKeyNameTipText()
```

:   Returns the tip text for this property

    :   **Returns:**: tip text for this property suitable for displaying in the explorer/experimenter gui

---


### getCacheKeyName

```
public java.lang.String getCacheKeyName()
```

:   Get the value of CacheKeyName.

    :   **Returns:**: Value of CacheKeyName.

---


### setCacheKeyName

```
public void setCacheKeyName(java.lang.String newCacheKeyName)
```

:   Set the value of CacheKeyName.

    :   **Parameters:**: `newCacheKeyName` - Value to assign to CacheKeyName.

---


### getRevision

```
public java.lang.String getRevision()
```

:   Returns the revision string.

    :   **Specified by:**: `getRevision` in interface `RevisionHandler` **Overrides:**: `getRevision` in class `DatabaseUtils`
    :   **Returns:**: the revision


---


|  |  |  |
| --- | --- | --- |
| **Overview** \| **Package** \| **Class** \| **Tree** \| **Deprecated** \| **Index** \| **Help** \| **Weka's home** | |  |
| **PREV CLASS**   **NEXT CLASS** | **FRAMES**    **NO FRAMES**     **All Classes** |  |
| SUMMARY: NESTED \| FIELD \| CONSTR \| METHOD | DETAIL: FIELD \| CONSTR \| METHOD |  |


---
